# Review on the health-promoting effect of adequate selenium status

**DOI:** 10.3389/fnut.2023.1136458

**Published:** 2023-03-16

**Authors:** Ying Sun, Zhineng Wang, Pin Gong, Wenbo Yao, Qian Ba, Hui Wang

**Affiliations:** ^1^School of Food and Biotechnological Engineering, Shaanxi University of Science and Technology, Xi’an, China; ^2^State Key Laboratory of Oncogenes and Related Genes, Center for Single-Cell Omics, School of Public Health, Shanghai Jiao Tong University School of Medicine, Shanghai, China

**Keywords:** inorganic selenium, organic selenium, selenium intake, health-promoting effects, selenium status

## Abstract

Selenium is an essential microelement involved in various biological processes. Selenium deficiency increases the risk of human immunodeficiency virus infection, cancer, cardiovascular disease, and inflammatory bowel disease. Selenium possesses anti-oxidant, anti-cancer, immunomodulatory, hypoglycemic, and intestinal microbiota-regulating properties. The non-linear dose-response relationship between selenium status and health effects is U-shaped; individuals with low baseline selenium levels may benefit from supplementation, whereas those with acceptable or high selenium levels may face possible health hazards. Selenium supplementation is beneficial in various populations and conditions; however, given its small safety window, the safety of selenium supplementation is still a subject of debate. This review summarizes the current understanding of the health-promoting effects of selenium on the human body, the dietary reference intake, and evidence of the association between selenium deficiency and disease.

## 1. Introduction

Selenium is an essential trace element for the human body that was discovered in 1817 by the Swedish chemist Berzelius (1). Numerous studies have demonstrated that selenium possesses anti-oxidant, anti-cancer, immunomodulatory, hypoglycemic, and intestinal microbiota-regulating properties (2–5). Selenium deficiency can result in diminished immunity and increased vulnerability to infections, such as human immunodeficiency virus (HIV) and hepatitis B infections. Long-term selenium deficiency increases the risk of diseases such as Kaschin–Beck disease (KBD), Keshan disease (KD), acquired immunodeficiency syndrome (AIDS), cancer, cardiovascular disease (CVD), and inflammatory bowel disease (IBD) (6). According the World Health Organization (WHO), selenium intake is inadequate in multiple countries, including India, Belgium, Brazil, the United Kingdom, France, Serbia, Slovenia, Turkey, Poland, Sweden, Germany, Spain, Portugal, Denmark, Slovakia, Greece, the Netherlands, Italy, China, Austria, and Ireland (7). Therefore, reasonable selenium supplementation is essential for the human body. However, the safe selenium intake level is limited and not well defined (8).

This review examines the classification, food sources, clinical diseases, health-promoting effects, and dietary reference intake of selenium, as well as its relationships with AIDS, cancer, CVD, and IBD (9–11). Further, we provide recommendations for selenium intake in various populations and for the resolution of health issues caused by selenium deficiency.

## 2. Selenium classification

**FIGURE 1:**
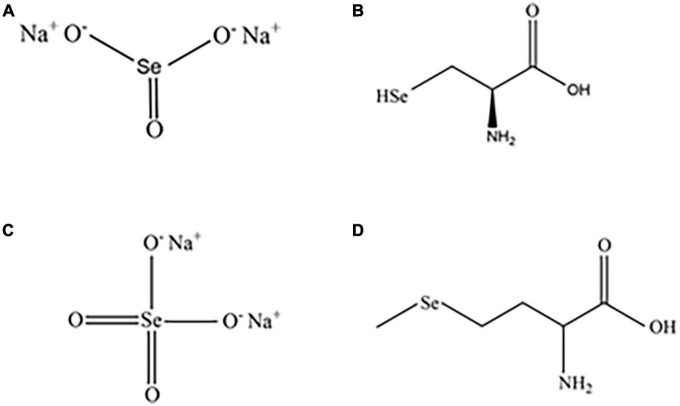
Structures of common inorganic and organic selenium compounds. Sodium selenite **(A)**, sodium selenate **(B)**, SeC **(C)**, and SeM **(D)**.

In nature, selenium exists in inorganic and organic forms. Inorganic selenium is obtained from metal deposit byproducts, primarily selenite (SeO_3_^2–^) and selenate (SeO_4_^2–^) (12, 13). Selenate and selenite are rare in nature and are typically complexed with sodium to form sodium selenite and sodium selenate, respectively (Figure 1) (14). Organic selenium is formed through the biotransformation of selenium and amino acids, primarily including selenomethionine (SeM) and selenocysteine (SeC) (Figure 1) (15).

As inorganic selenium is hardly absorbed by the human body and highly toxic, only a trace amount of this form is obtained from food (16). Organic selenium is more biocomand patible and more readily absorbed and stored in tissues than inorganic selenium, significantly improves the plasma selenium status in the human body. As a result, organic selenium exhibits greater biological activity and is therefore more widely used in supplement production (17, 18). For instance, when organic selenium is used as a selenium supplement for livestock, the selenium enrichment effect is greater than when inorganic selenium is used (15). Selenium is present primarily in the organic form in the majority of natural and selenium-rich foods. Selenium is primarily found in meat, eggs, bread, and fish (19, 20).

## 3. Environmental selenium exposure

### 3.1. Geographical distribution of selenium

Selenium is found in trace amounts in the Earth’s crust, typically in the range of 0.05–0.09 mg/kg. Soils can be classified into selenium-deficient (<0.13 mg/kg), selenium-marginal (0.13–0.18 mg/kg), selenium-sufficient (0.18–0.40 mg/kg), selenium-rich (0.40–3.00 mg/kg), and excess-selenium (>3.00 mg/kg) soils (21). Selenium is unequally distributed globally, with the Americas accounting for 52.7% of proven global selenium reserves, followed by Asia and Africa, which account for 15.4% each, Europe, which accounts for 12.2%, and Oceania, which accounts for 4.4% (22). While China’s selenium reserves are among the world’s largest and at present can meet the national selenium demand, the problem of unequal selenium resource distribution persists (23). Enshi, Hubei Province is dubbed the world’s selenium capital because of its widespread and large selenium resources and because it accommodates the world’s only independent selenium deposit. However, 22 provinces of China, accounting for 72% of the country’s territory, face selenium resource scarcity, with 30% being classified as severe selenium-deficient areas.

### 3.2. Form and distribution of selenium in foods

The amount of selenium in foods is highly variable and is influenced by the location of crops or the composition of the feed taken by animals. Bread, grains, meat, nuts, fish, eggs, and milk and other dairy products are major sources of selenium (20, 24).

The difference in selenium content between bread and cereals is 0.01–30 mg/kg, with the majority of selenium being in the forms of SeM (55–85%), SeC (4–12%), and selenate (25, 26). The selenium amounts of meat, fish, and eggs vary between 3 and 25 g, and the selenium concentration even varies among different sections of meat (19). Internal organs, particularly the liver and kidneys, contain comparatively high levels of selenium. For example, selenium concentrations in beef kidney, liver, and heart tissues are 4.5, 0.93, and 0.55 mg/kg, respectively, whereas muscle concentrations range between 0.2 and 0.55 mg/kg (27). In meat, selenium primarily exists as SeM (50–60%) and SeC. In fish, selenium contents typically range between 0.1 and 5.0 mg/kg, and selenium is primarily in the forms of SeM (29–70%) and selenite or selenate (12–45%) (28, 29). Milk contains selenium primarily in the forms of SeC and selenite. However, when selenium-enriched yeast is used to supplement selenium in milk from dairy cows, the type of selenium in the milk changes. Selenium is currently found mostly in the forms of SeC, SeM, and selenite (30). Fruits and vegetables also contain selenium, and vegetables cultivated in selenium-rich soils can enrich and transform the element. For instance, when onions, garlic, and broccoli are produced in selenium-rich soil, selenium levels can increase from <0.5 mg/kg to 140–300 mg/kg (31).

In addition to supplementation through selenium-enriched foods, selenium supplements are an efficient direct supplementation method. Selenium is currently available as multivitamin and multimineral supplements as well as stand-alone supplements, typically in the form of SeM, selenium-enriched yeast (grown on a selenium-rich medium), sodium selenite, or sodium selenate (23, 32, 33). Selenium-enriched yeast is the most common dietary source of selenium, primarily in the form of cysteine (34).

### 3.3. Selenium deficiency-related diseases

Selenium is a trace element that plays critical roles in human growth and development. It promotes human health by assisting in metabolism, boosting immunity, increasing physical fitness, and delaying aging. Selenium deficiency can impair body function and result in various diseases, including KBD, KD, neurological system disorders, and immunological deficiency disorders (35–38). Selenium deficiency can be diagnosed by measuring the serum or plasma selenium level, which should be at least 85 μg/L (39).

#### 3.3.1. KBD

KBD is an endemic, chronic, and degenerative osteoarthropathy that occurs in selenium-deficient parts of the world. It is the most prevalent in the diagonal zone extending from northeast to southwest China, but also occurs in Mongolia, Siberia, and Korea. It is a type of osteoarthritis characterized by cartilage tissue atrophy, degradation, and necrosis. It is the most prevalent in youngsters between the ages of 5 and 13 years. The primary signs include swollen joints, shortened fingers and toes, growth retardation, and stunting (35, 40). Patients with KBD have unusually low selenium levels in the hair and whole blood, and markedly decreased glutathione peroxidase levels in the blood (35, 40). A 0.1% sodium selenite aqueous solution is often used to treat children with KBD, with great results (41). A meta-analysis of 10 randomized controlled studies revealed the efficacy of selenium supplementation in the treatment of individuals with KBD; however, the data are limited by the possibility of bias (42). Zou et al.’s meta-analysis of KBD indicates that selenium supplementation is useful for preventing KBD in children (43). In a double-blind, randomized, controlled experiment, Moreno-Reyes et al. reported that supplementation with 100 g of selenium per day reduced clinical symptoms of KBD in children aged 5 to 15 years (44).

#### 3.3.2. KD

KD is a endemic cardiomyopathy that is prevalent in parts of China lacking in selenium. It is the most common in children between the ages of 2 and 10 years and in women of reproductive age. KD occurs across northeast to southwest China. The disease’s primary clinical manifestations include acute or chronic heart attacks marked by exhaustion, arrhythmia, and palpitation following limited exertion, inappetence, cardiac insufficiency, cardiac hypertrophy, and congestive heart failure. It is classified into four clinical subtypes: acute, subacute, chronic, and latent. Except for the latent form, case fatality rates are quite high. Pathological changes include numerous foci of cardiac necrosis and fibrosis. Ultrastructural examinations have revealed that membrane organelles, such as the mitochondria, and the sarcolemma are the first to be affected. The disease has a seasonal prevalence and can emerge as soon as three months following exposure to conditions that increase the risk of myocarditis (35, 45).

The mean hair selenium concentration in KD areas is <0.122 mg/kg, whereas it is >0.200 mg/kg in non-KD areas. The selenium concentrations in KD patients’ muscle, heart, liver, and kidneys are 10-fold lower than those in healthy people (35). The WHO recommends a minimum selenium intake of 21 mg/d for men and 16 mg/d for women to avoid KD development (46). Oral selenium is a very effective preventative strategy during the first three months of the KD risk period. Oral sodium selenite successfully prevents KD and considerably reduces its incidence rate (41). In a 10-year follow-up study of 302 patients with chronic KD and congestive heart failure, Zhu et al. showed that weekly supplementation with 1 mg of selenium decreased mortality (47). A comprehensive review and meta-analysis of Kawasaki disease indicate that selenium supplementation considerably lowers the incidence of Kawasaki disease (48).

Biochemical and clinical investigations have suggested that reduced glutaminase activity may decrease mitochondrial defense against peroxide-induced membrane damage and thus cause KD (36). KD is caused by selenium deficiency in association with coxsackie enterovirus infection. Inadequate selenium intake results in reduced selenoprotein anti-oxidant activity, and oxidative damage to viral DNA enhances its toxicity (49).

#### 3.3.3. Nervous system disease

Selenium is differentially distributed in various parts of the brain, with peak concentrations in gray matter-rich areas and glands (50). When a diet has insufficient selenium, brain selenium is retained in the organs, interfering with the normal supply route and resulting in the development of severe neurological dysfunction (51). Selenium plays a critical role in the brain and selenium deficit results in neurodegenerative diseases such as Alzheimer’s disease, Parkinson’s disease, and epilepsy (38, 52). Nervous system diseases can be avoided by supplementation with selenium-rich yeast. Selenium supplementation is effective in reducing intractable epileptic seizures in children (39). Cardoso et al. conducted a randomized, controlled study of 40 Alzheimer’s patients over a period of 24 weeks. The studies demonstrated that supplementation with 30 mg of selenium per day can enhance the selenium concentration in the central nervous system, halt neurodegeneration, and assist Alzheimer’s disease patients (53). In a clinical investigation, erythrocyte lipid peroxidation and glutathione peroxidase activity were greater in individuals with refractory epilepsy than in normal adults; nevertheless, supplementation with 200 g/day decreased lipid peroxidation, glutathione peroxidase activity, and morbidity in epileptic patients (54).

#### 3.3.4. Virus infection susceptibility caused by selenium deficiency

The development of early HIV infection has been associated with low plasma selenium levels. Subclinical malnutrition is a significant factor in the development of AIDS. However, there is compelling evidence that the magnitude of selenium deficiency is predictive of the occurrence of AIDS and associated mortality (37, 55). Selenium deficiency also enhances the toxicity of other RNA viruses, such as hepatitis B virus, and of hemolytic anemia. The underlying mechanisms are currently being investigated.

## 4. Human health-promoting effects of selenium

**FIGURE 2:**
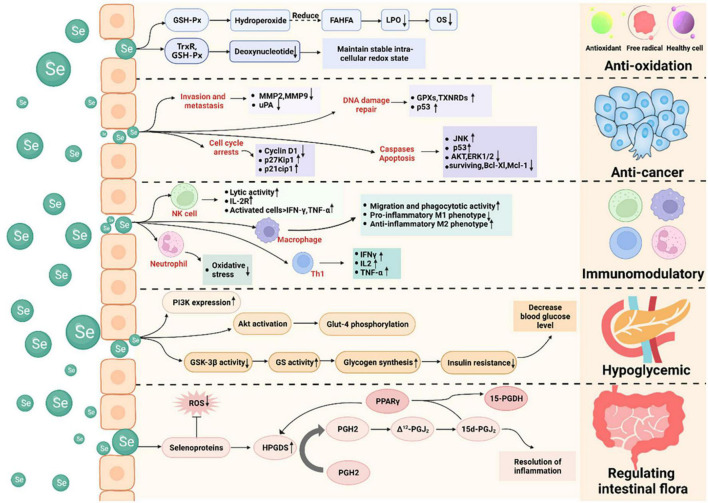
Health-promoting effects of selenium and its mechanisms of action. Selenium possesses anti-oxidant, anti-cancer, immunomodulatory, hypoglycemic, and intestinal microbiota-regulating properties. It acts as an anti-oxidant by lowering oxidative stress and deoxynucleotide levels. It functions as an anti-cancer agent by inducing apoptosis and cell-cycle arrest, preventing tumor cell invasion and metastasis, and promoting DNA damage repair. It exerts immunomodulatory effects by affecting non-specific immunity (e.g., macrophages and neutrophils) and specific immunity (e.g., T and B lymphocytes). It exerts hypoglycemic action by regulating the IRS-PI3K-Akt signaling pathway. It regulates the intestinal microbiota by regulating prostaglandins.

Selenium is an essential trace element in the human body, and supplementation with selenium has been shown to benefit human health. Selenium has anti-oxidant, anti-cancer, immunomodulatory, hypoglycemic, and intestinal microbiota-regulating properties, and its mechanisms of action have been investigated (Figure 2).

### 4.1. Anti-oxidant effects

When an organism is stressed or diseased, excess free radicals are generated and react with unsaturated lipids in the cell membranes, resulting in lipid peroxidation and severe damage to the biological system. Selenium is a component of glutathione peroxidase, which can convert hydroperoxide into hydroxy fatty acids, limiting lipid peroxidation by free radicals and lowering oxidative stress (56). It is widely recognized that selenium plays a direct or indirect role in removing intracellular free radicals (57, 58). Anti-oxidants prevent or mitigate oxidative DNA damage, and anti-oxidant enzymes require mineral cofactors, such as selenium for glutathione peroxidase and zinc and copper for superoxide dismutase (59, 60). Selenium functions as a redox center, protecting tissues from free radical-induced cell damage. Selenase and thioredoxin reductase are both capable of reducing nucleotides in deoxyribonucleic acid and thus maintain intracellular redox homeostasis (39).

An excess amount of reactive oxygen species (ROS) in the bloodstream causes DNA damage and oxidative stress in cells (61). Cells are predisposed to oxidative stress when their anti-oxidant contents are low or their ROS levels high. Selenium can prevent an overabundance of ROS, preserve the redox state of cells, and suppress oxidative stress (62). Fujieda et al. demonstrated that selenium deficiency results in a considerable decrease in glutathione peroxidase activity, which results in an increase in oxidative stress levels, and that sodium selenite treatment is efficient in ameliorating this condition (63). Plasma selenium levels are adversely correlated with oxidative stress levels in children with upper respiratory tract infections, patients with oral orofacial inflammatory disease, and pregnant women (64–66). However, Gać et al. measured plasma selenium concentrations, oxidative stress levels, and total anti-oxidant status in 337 children (mean age: 8.53 ± 1.92 years) and found that the plasma selenium concentration was not negatively correlated with oxidative stress levels, but was positively correlated with the total urine anti-oxidant status. Increased plasma selenium concentrations in healthy children have been shown to improve their overall anti-oxidant status (67). Thus, selenium exhibits anti-oxidant activity and suppresses oxidative stress, hence protecting the human body from oxidative stress-induced damage (4). Studies have demonstrated that high dosages of selenium can elicit cytotoxicity by elevating intracellular ROS, resulting in DNA damage and oxidative stress. High doses of selenium can also cause decreased immunological function and carcinogenic effects (68). Zachariah et al. studied the effects of high dosages of selenium on endothelial cells and reported that high doses of selenium inhibited NO bioavailability and angiogenesis. In addition to inducing ER stress and increasing the generation of ROS, selenium at high dosages can cause endothelial dysfunction (69). Consequently, selenium administration at high dosages induces oxidative stress, resulting in cytotoxicity and endothelial dysfunction. These findings highlight the significance and potential dangers of selenium supplementation as an antioxidant.

Owing to its anti-oxidant action, selenium is frequently employed in product development as a bioactive ingredient. Mileti et al. demonstrated that the addition of sodium selenite greatly improved DPPH clearance and Fe^2+^ chelation during exopolysaccharide synthesis (70). According to Xia et al., adding modest concentrations of selenium (0.5 and 1.0 mmol/L) increased the germination rate of alfalfa seeds and their superoxide dismutase, catalase, ascorbate peroxidase, and peroxidase activities, and decreased their malondialdehyde content (71). Forootanfar et al. reported that selenium-containing nanoparticles and selenium dioxide had DPPH radical-scavenging activities of 23.1 ± 3.4% and 13.2 ± 3.1%, respectively, at the same dose (200 μg/mL). However, findings from reduction capability measurements indicated that selenium dioxide has higher electron donor activity than selenium-containing nanoparticles (72). Xiao et al. developed nanoparticles containing selenium that exhibit strong anti-oxidant activity (73).

In conclusion, selenium, both organic and inorganic, exhibits anti-oxidant effects (63). The organic forms are selenoprotein and SeC, and selenoprotein has a critical physiological role in the body. Approximately 50% of all known selenoproteins have anti-oxidant properties (39). Inorganic selenium acts as an anti-oxidant by lowering oxidative stress and increasing DPPH-scavenging and Fe^2+^-chelating abilities (63).

### 4.2. Anti-cancer effects

The association between selenium and cancer has long been a source of controversy. However, in recent years, numerous studies have demonstrated the efficacy of selenium in suppressing carcinogenesis and enhancing immunity and anti-oxidant capacity.

Recently, there has been a surge of interest in the development of nanomaterials with increased anti-cancer activity and less adverse effects on the body as prospective cancer treatment options. In this light, selenium-containing nanoparticles are being investigated as potential cancer treatment agents because selenium is an essential trace element and nanomaterials containing selenium are more biocompatible. Selenium-containing nanomaterials have been found to have anti-ovarian cancer and anti-bone tumor properties. Toubhans et al. demonstrated that inorganic selenium nanoparticles triggered nanomechanical responses, changes in cell-surface roughness and membrane hardness, and cell apoptosis in SKOV-3 and OVCAR-3 ovarian cancer cells, indicating that selenium effectively inhibits the growth of ovarian cancer cells (2). Selenium-doped hydroxyapatite nanoparticles are frequently employed as bone-induction biomaterials. Barbanente et al. demonstrated that hydroxyapatite nanoparticles doped with selenium at low concentrations are biocompatible and may be used to treat bone cancers (74).

Selenium molecules in food undergo metabolic transformations via various pathways, producing a diversity of selenium metabolites with varying biological activities. Redox-active selenium metabolites have improved nucleophilic capabilities and high reactivity, making them powerful anti-cancer agents (75). At present, selenite is the most effective dietary selenium anti-cancer medication licensed by the United States Food and Drug Administration. When selenium is in the + 4 oxidation state as sodium selenite, it can react directly with the cysteine clusters found in the catalytic subunits of enzymes such as protein kinase C. Selenium compounds can oxidize the sulfhydryl groups in the catalytic domain of protein kinase C to disulfide bonds, inactivating the enzyme (76). This is because it oxidizes key thiol-containing enzymes and generates ROS. In addition, selenium compounds exert cytotoxic effects by acting as pro-oxidants, disrupting cellular redox homeostasis, and triggering selenium-induced apoptosis in mutant abnormal cells (57, 77).

Selenium promotes apoptosis, an important anti-cancer mechanism. Methylselenic acid (MSeA) has been found to increase caspase-mediated apoptosis by downregulating survivin, Bcl-xL, and Mcl-1 expression (78, 79). In LNCaP human prostate cancer cells, selenite induced p53 Ser-15 phosphorylation and caspase-mediated apoptosis (80). MSeA exposure induced caspase-mediated apoptosis in DU145 human prostate cancer cells, which was associated with reduced phosphorylation of protein kinase B (Akt) and extracellular regulated kinase ½ (81). MSeA-induced G1 arrest in DU145 cells was associated with increased p27kip1 and p21cip1 expression (82). Selenium induced cell-growth arrest and death *in vivo*, which was associated with decreased cyclin D1 expression, increased p27kip1 expression, and the activation of c-Jun NH2-terminal kinase (JNK) (83).

Inhibition of tumor cell invasion and metastasis is another important anti-cancer mechanism of selenium. Matrix metalloproteinase (MMP)-2 and MMP-9 degrade the extracellular matrix and basement membrane and play key roles in tumor invasion and metastasis. The urokinase-type plasminogen activator (uPA) system has been associated with tumor invasion, metastasis, and decreased patient survival time (84). Selenite inhibits tumor cell invasion by inhibiting the expression of MMP-2, MMP-9, and uPA (85).

Stimulating DNA damage repair also is an important anti-cancer mechanism of selenium. Given the critical role of selenoproteins (such as glutathione peroxidases and thioredoxin reductases) in anti-oxidant defense and maintaining a reducing cell environment, selenium can accelerate the DNA damage repair response by enhancing selenoprotein production (86). SeM boosts p53 activity and protects cells from DNA damage via its anti-oxidant activity (87). However, Duffield-Lillico et al. discovered in a double-blind, randomized, placebo-controlled clinical study that selenium supplementation did not prevent basal cell carcinoma but increased the incidence of squamous cell carcinoma and non-melanoma skin cancer (82). Algotar et al. observed in a 5-year double-blind, randomized, placebo-controlled experiment that selenium intake of 200 or 400 μg per day increased the incidence of non-melanoma skin cancer (88). In a major clinical research study including 5,345 men, Kristal et al. reported that supplementation with selenium raised the risk of prostate cancer by 91% in men who previously ingested appropriate quantities of selenium (89). In a 22-year follow-up analysis of 4,459 patients with non-metastatic prostate cancer, Kenfield et al. reported that selenium supplementation of 140 μg or more per day may increase prostate cancer mortality (90). Thus, supplementation with selenium raises the incidence of squamous cell carcinoma, non-melanoma skin cancer, and high-grade prostate cancer (91).

In conclusion, selenium functions as an anti-cancer agent by triggering apoptosis and cell-cycle arrest, preventing tumor cell invasion and metastasis, and promoting DNA repair. The anti-cancer effects of selenium in colon, skin, breast, liver, lung, and rectal cancers have since long been documented. Selenium has great clinical potential as an anti-cancer agent (2, 3, 92–96). However, selenium supplementation raises the risk of squamous cell carcinoma, non-melanoma skin cancer, and high grade prostate cancer (91).

### 4.3. Immunomodulatory effects

The immune system is the most effective barrier against pathogen invasion. It recognizes and eliminates antigenic foreign substances and cooperates with other body systems to preserve homeostasis and physiological balance. Natural killer (NK) cells are vital immune cells that are involved in anti-tumor, anti-viral infection, and immunological control functions. Selenium is required for the regular functioning of the immune system and can affect non-specific immunity (e.g., macrophages and neutrophils) and specific immunity (e.g., T and B lymphocytes). Selenium deficiency results in immune system dysfunction, which harms immunological function. Broome et al. demonstrated that selenium supplementation raised plasma selenium levels, the body’s exchangeable selenium pool, lymphocyte phospholipids, and cytosolic glutathione peroxidase activity. Selenium supplementation boosts cellular immune responses and the expression of cytokines by enhancing interferon secretion and increases early peak T cell proliferation and T helper cell counts. Subjects supplemented with selenium exhibited quick poliovirus elimination and the reverse transcriptase-PCR products of polioviruses recovered from their feces had a low number of mutations (97). Selenium supplementation promotes lymphocyte proliferation in response to mitogens, increases the expression of high-affinity IL-2 receptors, and enhances tumor cytotoxicity and NK cell activity mediated by cytotoxic lymphocytes (97, 98). Selenium supplementation has also been found to boost NK cell activity, T cell proliferation, lymphokine-activated killer cell activity, delayed onset of cutaneous allergy reactions, and vaccine-induced immunity in experimental animals (5).

Selenoprotein is thought to play a role in the epigenetic control of pro-inflammatory genes. Narayan et al. demonstrated that selenium supplementation decreased histone H4 acetylation at K12 and K16 in the *COX-2* and *TNF*-α promoters and of the p65 subunit of the redox-sensitive transcription factor nuclear factor kappa B in primary and immortalized macrophages, indicating the critical role of selenoprotein in inhibiting histone H4 acetylation (99). T cell acute lymphoblastic leukemia/lymphoma is a chemotherapy-sensitive hematologic malignancy. Wu et al. demonstrated that ethylene glycol selenoprotein-induced apoptosis in T cell acute lymphoblastic leukemia/lymphoma cells is mediated by caspase activation and increased ROS via the activation of mitochondrial signaling pathways (100). Jiang et al. demonstrated that selenium-enriched chitosan oligosaccharide effectively enhanced phagocytosis, anti-inflammatory cytokine secretion in peritoneal macrophages, phagocytotic, spleen, and thymus indices, and immunity, with no obvious toxicity, in Kunming mice (96). Albumin acts as a carrier of nutrients, whereas globulin is an immunoprotein. The albumin-to-globulin ratio in serum is a useful indicator of animal nutrition and immunological function (101). Interleukin (IL)-2 is a component of cellular immune responses and a critical immunological regulator, regulating cell development, differentiation, and proliferation and contributing to the resolution of viral or bacterial infection. In laying hens, a selenium-enriched earthworm powder containing 1.0 mg/kg selenium increased albumin, globulin, immunoglobulin G, and IL-2 expression levels (101).

Macrophages of the M2 phenotype produce anti-inflammatory cytokines, such as IL-10, which suppress tumor development (102). Selenium supplementation improved migratory and phagocytic activities in selenium-deficient macrophages and promoted the transition from the pro-inflammatory M1 phenotype to the anti-inflammatory M2 phenotype, thereby lowing pro-inflammatory action (103). Selenium supplementation also provides protection against endogenous oxidative stress in neutrophils (104). In the elderly, increased serum selenium levels are positively associated with an increase in the number and activity of NK cells. Selenium has been shown to boost the expression of the IL-2 receptor on the NK cell surface, thus increasing the proliferation and clonal expansion of cytotoxic precursor cells and the lytic activity of activated NK cells (105). Activated NK cells exhibit cytotoxicity toward tumor cells and release immunoregulatory molecules such as IFN-γ and TNF-α (106). Selenium supplementation has been shown to have an effect on T cell activation and function (107). For example, a selenium-rich diet can shift the balance of T helper 1/T helper 2 cells toward the T helper 1 phenotype and increase IFN and CD40 ligand levels (108). However, Ivory et al. have shown that selenium administration increases IL-10 release and decreases CD8 T cell granzyme B levels in the blood (91, 109). Not only do perforin and granzyme destroy virus-infected cells and tumors, but they also modulate the immune response to viral infections (110). Immune modulation is impacted both positively and negatively by selenium administration, as demonstrated by the preceding results.

In conclusion, selenium can protect neutrophils from endogenous oxidative stress, increase the migratory and phagocytic activity of macrophages and promote the anti-inflammatory M2 type, and increase the lytic activity of NK cells in order to exert immunomodulatory effects. Selenium can also exert an immunomodulatory effect through the recruitment of T helper 1 cells and the release of pro-inflammatory cytokines. However, selenium can also diminish the number of CD8 T cells and granzyme B, which impacts the control of the immune system.

### 4.4. Hypoglycemic effects

Diabetes mellitus is a chronic metabolic endocrine disease that affects a large proportion of the global population. Diabetes affects approximately 425 million adults worldwide and this number has been projected to increase to 629 million by 2045 (111). Serum selenium levels do not appear to be related with newly diagnosed type 2 diabetes in humans, although they are considerably increased in individuals with type 2 diabetes. Selenium supplementation has been shown to increase the incidence of type 2 diabetes in elderly people, particularly men with high baseline selenium levels, but not in the general population (112, 113). However, a recent high-quality randomized controlled study showed that supplementation with selenium (200 μg/d) in the form of selenide yeast or L-selenomethionine had no effect on the incidence of type 2 diabetes (114). Therefore, it has been suggestion that increased selenium consumption may be associated with an increased risk of developing diabetes (115). In a study involving in 41,474 subjects, Lin et al. found that dietary selenium intake was positively associated with increased plasma glucose and glycosylated hemoglobin levels, as well as the risk of developing diabetes (116). Additionally, they observed a positive association between serum selenium levels and increased plasma glucose and glycosylated hemoglobin levels. This supports the notion that elevated plasma selenium levels are related with an increased risk of developing diabetes (117, 118). This is primarily because high selenium intake increases the expression of peroxisome proliferator-activated receptor coactivator (PGC-1), a transcriptional coactivator involved in cellular energy metabolism, which may be one of the primary causes of hyperglycemia associated with high selenium intake (119).

However, appropriate selenium supplementation is a critical component in controlling glucose homeostasis in humans (120). El-Borady et al. demonstrated that selenium nanoparticles can help prevent hyperglycemia by lowering plasma glucose levels. Selenium nanoparticles also enhanced insulin levels in the plasma and pancreas of diabetic rats and repaired damaged pancreatic tissue. Additionally, selenium nanoparticles reduced oxidative stress at the transcriptional and cellular levels and enhanced glutathione peroxidase activity (111). Chen et al. demonstrated that supplementing diabetic rats with selenium normalized the glucose-6-phosphatase, lactate dehydrogenase, and glycogen phosphorylase activities and restored glycogen levels to their pre-diabetic levels. Selenium supplementation may enhance glucose uptake and metabolism in the liver by regulating glucose metabolic enzyme activity and mediating insulin-like actions in diabetes (121).

Selenium polysaccharide has a substantial hypoglycemic effect as a particular target of the IRS-PI3K-Akt signaling pathway (122). Polysaccharides may have several hypoglycemic mechanisms. First, polysaccharides have been shown to increase PI3K expression. Second, selenium polysaccharide has been shown to activate Akt and phosphorylate Glut-4. Third, selenium polysaccharide may inhibit GSK-3 action, thus increasing glycogen synthesis and boosting glycogen synthesis (123). However, Zhou et al. have shown that long-term feeding of mice, rats, and pigs with a high-selenium diet (0.4–0.30 mg/kg diet) results in hyperinsulinemia, hyperglycemia, insulin resistance, and glucose intolerance (124). Several studies have demonstrated that a high selenium intake may enhance the activity of GPx1 and other selenoproteins, thereby altering the function of major regulators of glycolysis, gluconeogenesis, and fat synthesis, thereby increasing the prevalence of diabetes (125–130). Ogawa-Wong et al. have demonstrated an increased incidence of type 2 diabetes in individuals with high selenium levels at baseline. Therefore, long-term supplementation with high doses of selenium increases the likelihood of diabetes, and selenium supplementation may have detrimental effects on those who already have adequate selenium levels (131).

In conclusion, selenium has potential as a medicine in the treatment of diabetes, but the optimal dose of selenium requires additional research.

### 4.5. Regulation of the intestinal microbiota

Intestinal microbes significantly contribute to human physiology by regulating the maturation and proliferation of intestinal cells, aiding food digestion, protecting against harmful bacteria, and regulating the intestinal mucosal immune response (132, 133). Dietary components, particularly trace elements, can affect the colonization of the gastrointestinal tract and the makeup of the microbiota structure. Selenium supplementation increases the diversity of the microbial community and has various effects on different microbiota categories. Thus, selenium has a unique role in many microbiota (134).

Selenium shows specific antibacterial activity against pathogenic bacteria such as *Escherichia coli* in the complex context of the cecal microbiota, without affecting the abundance of other community members (135). Lin et al. demonstrated that selenium administration improved the diversity and relative abundance of intestinal microbes, restored some intestinal microbiota, and increased methylmercury breakdown and excretion in rats exposed to methyl mercury (136). Approximately a fifth of the intestinal microbiota is capable of expressing selenoprotein, and selenium availability affects selenoprotein expression (137). Selenoproteins are required for various activities in both bacteria and mammalian hosts (138). Dietary selenium has an effect on the composition of the intestinal microbiota and gastrointestinal tract colonization, which in turn affects the host’s selenium status and selenoprotein expression (137). Takahashi et al. demonstrated that selenium-methyl SeC and selenocyanate are converted to selenomethionine by intestinal bacteria, indicating that selenium compounds can be converted to selenomethionine by the microbiota and subsequently utilized by the host (134). Using 16S rRNA gene amplicon sequencing to analyze bacterial communities and microbial metabolic pathways, Kang et al. found that the administration of selenium-enriched *Lactobacillus plantarum* significantly increased the metabolization of selenocysteine, selenocystathionine, and selenomethionine, as well as plasma selenium levels and anti-oxidant capability in mice (139).

Selenoprotein affects the intestinal microbiota and increases the expression of hematopoietic PGD_2_ synthase (HPGDS), which catalyzes PGD_2_ synthesis in immune cells such as macrophages and T cells. PGD_2_ dehydrates and isomerizes spontaneously to create prostaglandins J_2_ (Δ^13^ -PGJ_2_) and Δ^12^-PGJ_2_, respectively, and Δ^12^-PGJ_2_ can be transformed into 15-deoxy-Δ^12,14^-prostaglandin J_2_ (15d-PGJ_2_) to alleviate inflammation. As ligands for the transcription factor peroxisome proliferator-activated receptor-γ (PPARγ), PGD_2_ metabolites can bind to PPAR-response elements in the *HPGDS* promoter and upregulate its expression, forming a feed-forward loop (140).

In conclusion, selenium can regulate the intestinal microbiota by controlling various prostaglandins.

## 5. Effects of selenium in different populations

**FIGURE 3:**
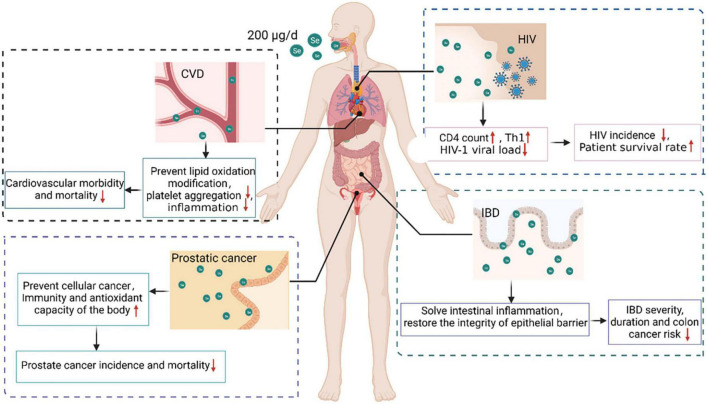
Beneficial effects and mechanisms of selenium supplementation in various diseases. Selenium supplementation can provide protection for populations with AIDS, cancer, CVD, and IBD. Supplementing HIV-infected people with 200 μg selenium per day enhanced the CD4 count, stimulated CD4 + T cell differentiation into T helper 1 cells, and decreased the HIV-1 viral load, thus decreasing HIV incidence and enhancing patient survival. Supplementing prostate cancer patients with 200 μg of selenium per day prevented cell carcinogenesis, enhanced the body’s immunity and anti-oxidant capacity, and reduced the incidence and mortality of prostate cancer. In a CVD population, supplementation with 200 μg selenium per day reduced lipid oxidation, platelet aggregation, and inflammation, thus decreasing CVD morbidity and mortality. Supplementing an IBD population with 200 μg of selenium per day relieved intestinal inflammation and restored epithelial barrier integrity, thereby decreasing the incidence, severity, duration, and risk of IBD-associated colon cancer.

The WHO recommends a selenium consumption of 34 μg/d for men and 26 μg/d for women, taking into account sex and bodyweight differences (141). According to the most recent report on dietary selenium reference intake in China, the recommended daily intake of selenium varies slightly across populations (23). An in-depth study in China and elsewhere revealed that women are deficient in selenium during pregnancy and lactation. Increasing the dietary selenium intake in pregnant and nursing women can successfully prevent miscarriage and reduce fetal teratogenicity (142). International attention has been focused on selenium intake, and recommended selenium intake standards for various populations have been established (Table 1) (141, 143–146). Low serum selenium status has been associated with disease risk (Table 2). Diseased populations benefit from a moderate selenium intake, and the ingested dose and action mechanism have been investigated (Figure 3).

**TABLE 1:** Recommended dietary selenium intakes for various regions and according to the WHO.

Age (years)/pregnancy status	WHO (μg/d) (141)		Northern Europe (μg/d) (144)		Japan (μg/d) (143)		China (μg/d) (145)	USA (μg/d) (146)
	**Women**	**Men**	**Women**	**Men**	**Women**	**Men**		
0–0.5	6		–		–		–	–
0.5–1	10		15		–		–	–
1–3	17		20		8	9	25	20
4–6	22		25		10	10	30	30
7–9	21		30		15	15	40	30
10–13	26	32	35	40	20	22.5	55	40
14–17	26	32	40	50	20	30	60	55
18–49	26	34	40	50	20	32.5	60	55
50–64	26	34	40	50	20	30	60	55
65–79	25	33	40	50	20	30	60	55
≥80	25	33	40	50	20	30	60	55
Pregnant women	28-30	–	55	–	24	–	66	60
Lactating women	35-42	–	55	–	–	–	78	70

“–” indicates that no data are available.

**TABLE 2:** Relationship between serum selenium status and disease risk.

T	Serum Se concentration (μ g/L)	Effect
HIV	≤85	Increased HIV morbidity and mortality (39, 148)
	–	
Cancer	<130	All-cause mortality and increased cancer mortality (154)
	>150	
Prostate cancer	<130	Increased incidence and mortality of prostate cancer (154, 157)
	>170	
CVD	≤55	Increased morbidity and mortality from CVD (167)
	>145	
IBD	ND	Increased incidence, severity, duration and colon cancer risk of IBD (169, 171)
	ND	

“–” indicates that no data are available.

### 5.1. Effects of selenium in HIV-positive patients

AIDS damages the immune system. It impairs immune function by destroying the human immune system’s most vital cells, CD4 + T lymphocytes. As a result, HIV-infected individuals are susceptible to various diseases, are at an increased risk of developing malignant tumors, and have a high mortality rate (147).

Selenium is required for normal immune system function and is an essential nutrient for AIDS patients. Selenium deficiency can result in immune system dysfunction, leading to reduced immunological function. Low plasma selenium levels are a strong predictor of HIV infection prognosis, and the degree to which plasma selenium levels drop is predictive of HIV incidence and mortality (6, 7).

CD4 count declines have been associated with decreases in plasma selenium levels in more than 20 publications. HIV patients who are selenium-deficient are 20 times more likely to die of HIV-related causes than those who have sufficient selenium (39). Selenium deficiency is defined as a plasma concentration of selenium that is less than or equal to 85 μg/L (148). Selenium promotes the differentiation of CD4 + T cells into T helper 1 cells, which is related with a decrease in the incidence of hospitalizations for coinfection in HIV-positive individuals (149). Baum et al. conducted a 24-month randomized, placebo-controlled study of 878 HIV-positive people who had never received antiretroviral treatment (150). The study findings indicated that taking a daily multivitamin and 200 μg of selenium in the early stages of HIV disease greatly decreased the risk of immunological decline and the incidence of HIV-related events. In a double-blind, randomized, placebo-controlled study in 450 adult male and female HIV patients, Hurwitz et al. found that nine months of selenium supplementation successfully enhanced blood selenium levels, prevented HIV-1 viral load progression, indirectly improved CD4 counts, decreased morbidity, and improved survival rates (151). Kamwesiga et al. undertook a 24-month, multicenter, double-blind, placebo-controlled, randomized clinical study, including 300 adult HIV patients. The results indicate that supplementation with 200 g of selenium per day can dramatically slow the pace of CD4 cell count reduction in HIV patients (152). In conclusion, supplementation with 200 g of selenium per day can minimize the risk of impaired immunity and the incidence of HIV and enhance the survival rate of HIV patients.

### 5.2. Effects of selenium in cancer patients

Cancer is a major public health problem worldwide and the second leading cause of death. Each year, an estimated 18.1 million new cases of cancer and 9.6 million cancer deaths occur globally (153). As a result, cancer imposes a significant global economic burden. Current clinical cancer treatments are inadequate in terms of effectiveness and biocompatibility. The association between selenium and cancer has long been a source of debate in the human health field. In recent years, extensive research has been conducted to demonstrate the efficacy of selenium in suppressing cellular carcinogenesis and enhancing the immune system and the body’s anti-oxidant capacity.

In a representative sample of the US population, non-linear relationships between serum selenium levels and all-cause and cancer mortality were observed; a negative association was observed at low selenium concentrations (<130 μg/L), whereas a moderate positive correlation was observed at high selenium concentrations (>150 μg/L) (154). The SELECT study revealed that selenium supplementation is related with an increased risk of prostate cancer in men with high baseline selenium levels (122). Supplementation of selenium in selenium-deficient people has been demonstrated to lower the risk of prostate cancer. In a seven-year, double-blind, randomized, placebo-controlled study in 32,400 man, Lippman et al. found that daily supplementation of 200 μg of selenium did not lower the incidence of prostate cancer in men who already consumed an adequate amount of selenium (155). A randomized controlled study by Duffield-Lillico et al. revealed that daily supplentation of 200 μg of selenium per day considerably decreased the incidence of prostate cancer in men with baseline selenium concentrations < 123.2 μg/L (156). Hurst et al. conducted a meta-analysis of blood selenium levels and non-linear dose-response relationships in 13,254 subjects with and 5,007 cases of prostate cancer and found that increasing serum selenium to 170 μg/L lowered the incidence of prostate cancer (157). Thus, selenium supplementation will help reduce cancer incidence and mortality in individuals who are deficient in selenium or have a minor deficiency (158). However, selenium supplementation is harmful to people with enough selenium and increases cancer incidence and death in individuals with high baseline selenium levels.

### 5.3. Effect of selenium in CVD patients

CVD is the leading cause of death worldwide (159). The burden of CVD is expected to increase with the aging population. Aging, smoking, obesity, elevated cholesterol levels, unhealthy eating habits, level of education, blood pressure, diabetes, and genetics have an effect on the risk of CVD (160).

Selenium has been shown to protect against CVD by suppressing lipid oxidation, platelet aggregation, and inflammation (161, 162). A study on the effect of long-term selenium yeast (200 μg/d) and coenzyme Q10 supplementation on cardiovascular mortality in elderly Swedes revealed that supplementation protected the heart in those with low baseline selenium levels (≤ 85 μg/L), but had no effect in those with plasma selenium levels > 85 μg/L (163). In a 12-year randomized, placebo-controlled study in 443 elderly subjects in good health, Alehagen et al. found that daily intake of 200 mg coenzyme Q10 and 200 μg selenium for four years significantly reduced cardiovascular mortality, and cardiovascular mortality was still decreased by more than 40% eight years after the four-year intervention (164). Yin et al. examined vitamin intake in 39,757 American adults using dietary recall data and found that consuming 207.8 μg selenium daily lowered CVD incidence and mortality (165). Additionally, a negative relation has been observed between selenium and total CVD through weighted quantile sum regression analysis (166). A meta-analysis of prospective observational studies revealed a non-linear relation between CVD risk and plasma selenium concentrations between 30–165 μg/L, but a substantial negative correlation in the range 55–145 μg/L; thus, the relation between baseline selenium status and the incidence of CVD may be non-linear and U-shaped (167). A meta-analysis of randomized controlled trials revealed that daily supplementation of 200 μg of selenium significantly enhanced blood selenium concentrations, whereas daily supplementation of 100 μg had no effect on CVD (167).

In conclusion, persons with low baseline selenium levels may benefit from supplementation, and supplementation with 200 g of selenium per day may reduce CVD morbidity and mortality; however, the preventive effect of selenium against CVD has not been demonstrated. To establish the association between selenium and CVD, larger clinical trials in populations with varying selenium levels are required. Future research should take into account the importance of selenium status, dosing, and safety.

### 5.4. Effect of selenium in IBD patients

IBD refers to a specific type of chronic inflammatory illness of the intestine, mostly including Crohn’s disease and ulcerative colitis (168). The incidence of IBD has been increasing over the last decades, mainly due to nutritional and environmental imbalances (169).

Selenocysteine is a selenoprotein involved in the regulation of inflammation (140). Serum selenium levels have been found to be decreased in patients with IBD (169). In New Zealand, the incidence rate of Crohn’s disease is among the highest and the mean plasma selenium levels among the lowest in the world (170). Serum selenium levels have been demonstrated to be adversely associated with the severity and length of IBD and the risk of colon cancer, and selenium may serve as a non-invasive biomarker of IBD activity and severity (171). Additionally, dietary selenium has been shown to be beneficial at resolving intestinal inflammation and reestablishing epithelial barrier integrity (140, 172). Selenium supplementation decreased colitis-associated inflammation and enhanced mouse survival in mice treated with dextran sodium sulfate (173).

In summary, low plasma selenium levels are associated with an increased risk of IBD. Selenium supplementation may help patients with IBD resolve their intestinal inflammation. The causative link between selenium deficiency and IBD requires further investigation.

## 6. Conclusions and future perspectives

Numerous studies have established that selenium possesses anti-oxidant, anti-cancer, blood glucose-lowering, and immune system-strengthening properties. Selenium supplementation benefits human health in various ways, most notably in terms of immunological responses and cancer prevention. Selenium supplements can be used to treat conditions such as HIV, IBD, CVD, and cancer. Selenium supplementation is most often accomplished through the use of inorganic sodium selenite, organic selenium, selenium nanoparticles, or selenium-enriched yeast. However, the relationship between selenium and human health is complex, and its “duality” makes research on its health effects difficult. Additionally, the non-linear dose-response relationship between selenium status and health is U-shaped; individuals with low baseline selenium levels may benefit from supplementation, whereas those with acceptable or high selenium levels may experience detrimental effects. Selenium has an extremely narrow range between deficiency and toxicity, and baseline selenium levels vary among populations. Safe methods and doses of selenium intake and the baseline selenium range suited for selenium supplementation remain to be established in future.

## Author contributions

YS and ZW drafted the manuscript. PG, WY, QB, and HW drafted and revised the manuscript. All authors contributed to the article and approved the submitted version.
