# Supplementary figures and images for: Review on the health-promoting effect of adequate selenium status
Source: Front Nutr. 2023 Mar 16;10:1136458. doi: 10.3389/fnut.2023.1136458 (PMC10060562; doi:10.3389/fnut.2023.1136458)

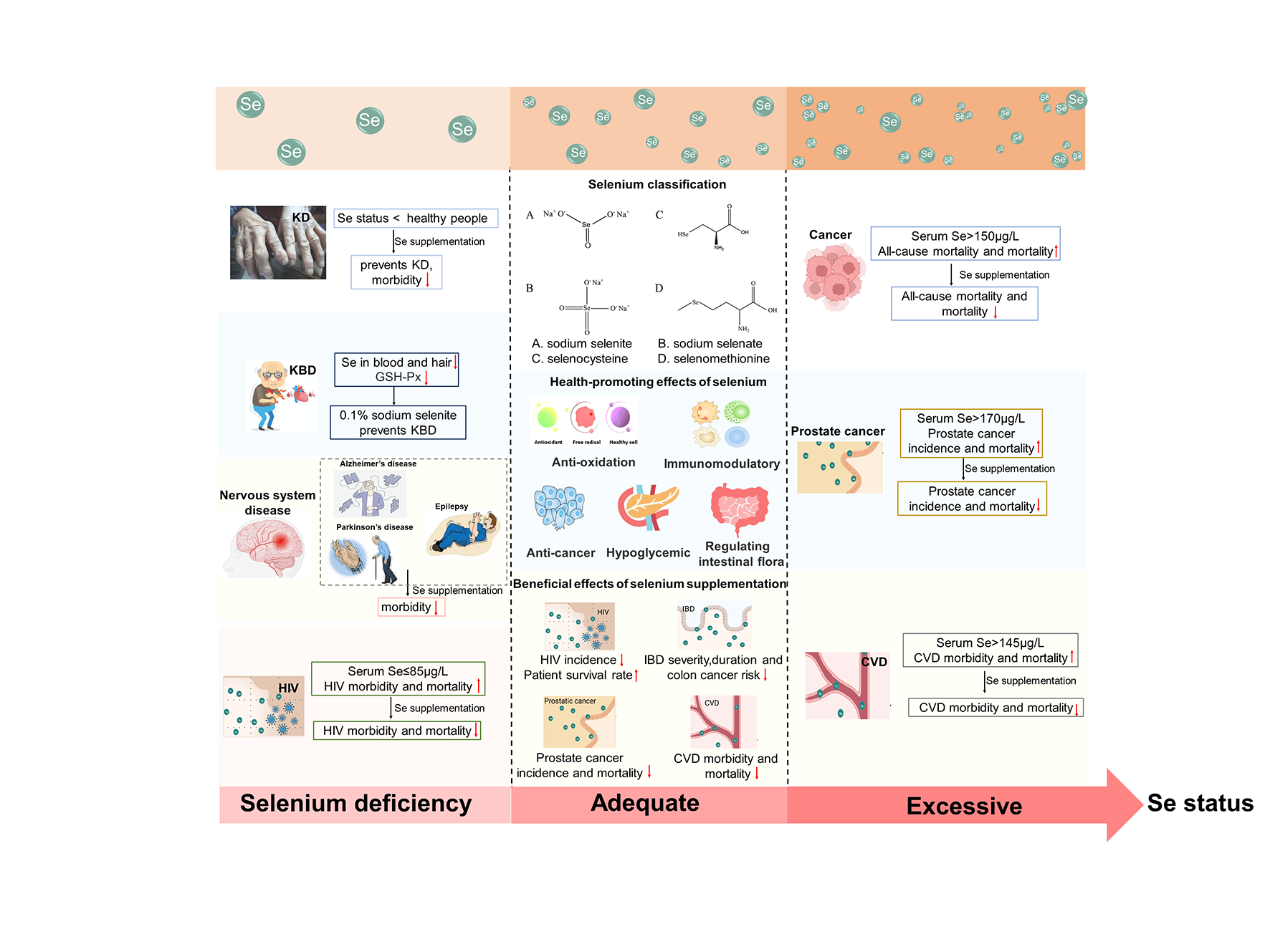

Supplement: Supplementary file 1 [file Image_1.TIF]
